# Supplementary material for: The Role of Uniform Meropenem Usage in Acinetobacter baumannii Clone Replacement
Source: Antibiotics (Basel). 2021 Jan 29;10(2):127. doi: 10.3390/antibiotics10020127 (PMC7911629; doi:10.3390/antibiotics10020127)
Supplement: Supplementary file 1 [file antibiotics-10-00127-s001.pdf]

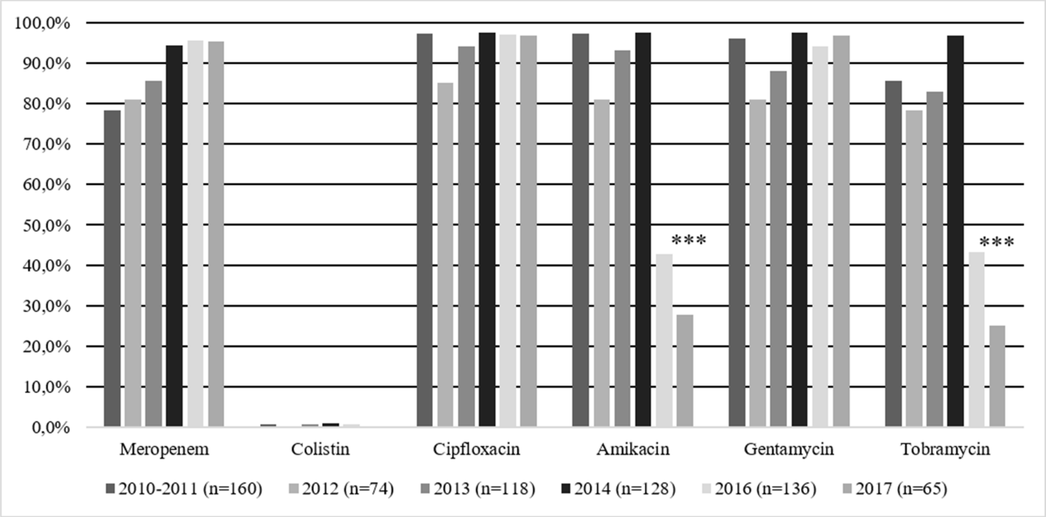

Figure S1. Results of the Kirby-Bauer disk diffusion tests, based on EUCAST recommendation. Significance levels: *→$p < 0.05$; **→$p < 0.01$; ***→$p < 0.001$.

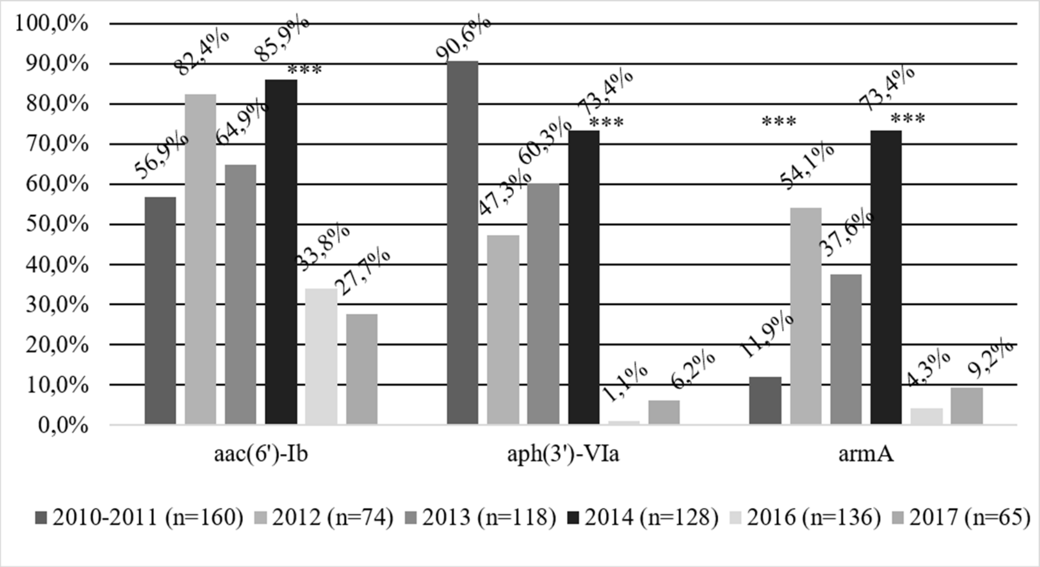

Figure S2. The prevalence of aminoglycoside resistance genes among the *A. baumannii* isolates by year. Significance levels: *→$p < 0.05$; **→$p < 0.01$; ***→$p < 0.001$.

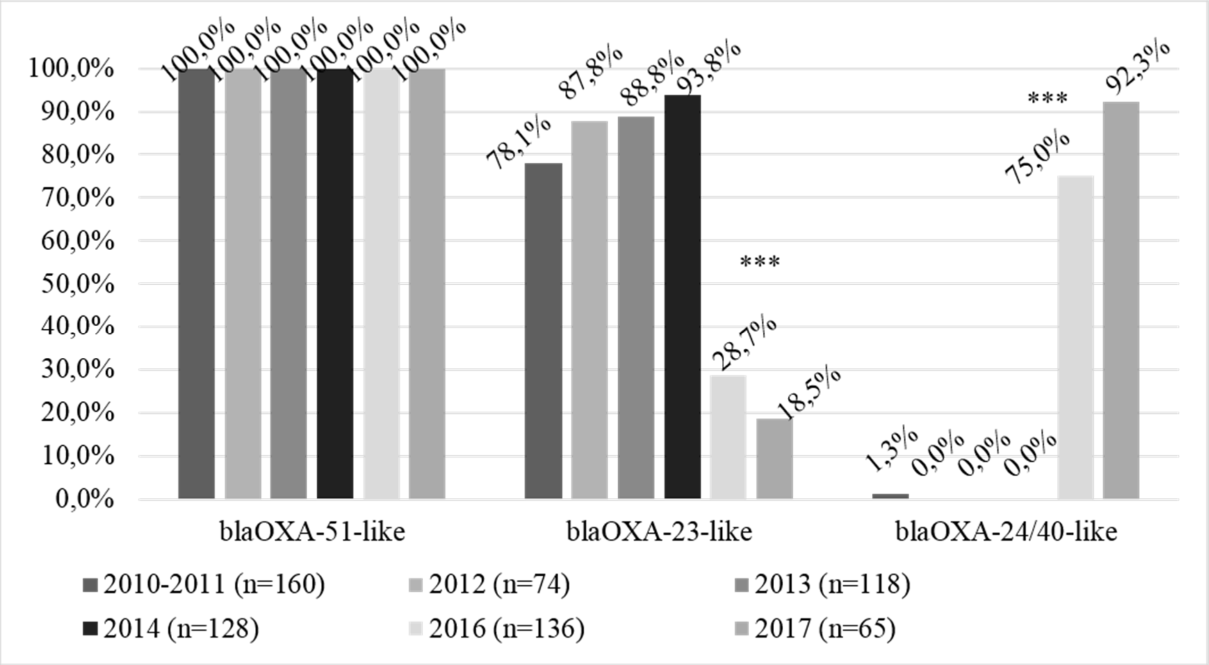

Figure S3. The prevalence of carbapenemases among the *A. baumannii* by years. Significance levels: *→$p < 0.05$; **→$p < 0.01$; ***→$p < 0.001$.
